# Role of Neoadjuvant Chemotherapy in Locally Advanced Carcinoma Stomach: An Analysis of the Short-Term Outcomes

**DOI:** 10.7759/cureus.23936

**Published:** 2022-04-07

**Authors:** Sooryabhala Sivacoumarane, Souradeep Dutta, Biswajit Dubashi, Subathra Adithan, Pampa C Toi, Vishnu Prasad Nelamangala Ramakrishnaiah

**Affiliations:** 1 Surgery, Jawaharlal Institute of Postgraduate Medical Education and Research, Puducherry, IND; 2 Medical Oncology, Jawaharlal Institute of Postgraduate Medical Education and Research, Puducherry, IND; 3 Radiodiagnosis, Jawaharlal Institute of Postgraduate Medical Education and Research, Puducherry, IND; 4 Pathology, Jawaharlal Institute of Postgraduate Medical Education and Research, Puducherry, IND

**Keywords:** short term outcomes, locally advanced gastric cancer, gastrectomy, neoadjuvant chemotherapy, carcinoma stomach

## Abstract

Introduction: Neoadjuvant chemotherapy (NACT) in carcinoma stomach was introduced in an effort to eliminate micro-metastasis and to improve resectablity before surgery which improves R0 resection rates. We aimed to study the short term outcomes of neoadjuvant chemotherapy on the Tumor Node Metastasis (TNM) stage and the operative outcomes including R0 resection rate in locally advanced gastric cancer.

Methods: We prospectively included patients with locally advanced adenocarcinoma stomach staged by contrast-enhanced computed tomography (CECT) in our study. Patients in Group I were started on neoadjuvant chemotherapy (epirubicin, oxaliplatin, and capecitabine). Surgery was done following response assessment CECT. Patients in Group II underwent upfront surgery. We assessed R0 resection rate, number of harvested and metastatic lymph nodes, lymph node ratio, duration of surgery, blood loss, hospital stay and complications between two groups. Response to NACT was assessed in Group I.

Results: Out of 47 patients who received NACT, two patients had complete response (4.2%), 13 had partial response (27.7%), 10 had stable disease (21.3%) and 22 patients had progressive disease (46.8%). We found no significant difference in the rate of R0 resection between the two groups (88.2% in NACT group vs 85.1% in surgery group, P=0.55).

Conclusions: The rate of R0 resection does not significantly improve with neoadjuvant chemotherapy. In view of high progression rates, patient selection is required when NACT is planned in carcinoma stomach which are surgically resectable at presentation. We await survival analysis to further validate the role of NACT.

## Introduction

The treatment of gastric cancer is curative resection in the form of D2 gastrectomy [1-3]. But more than 30% of gastric cancers present as locally advanced disease, making them unresectable [4,5]. Neoadjuvant chemotherapy (NACT) was introduced in an effort to eliminate micro-metastasis and to improve resectability before surgery which improves R0 resection rates.

Over the years several trials were conducted in gastric adenocarcinomas to evaluate the efficacy of NACT in improving overall survival [6-8]. The National Comprehensive Cancer Network (NCCN) recommends preoperative chemotherapy for locally advanced gastric cancers [9,10]. The most notable landmark trial is the Medical Research Council Adjuvant Gastric Infusional Chemotherapy (MAGIC) trial which compared perioperative chemotherapy followed by surgery and surgery alone in gastroesophageal (GE) adenocarcinomas [11]. These studies have demonstrated variable results in R0 resection rates but with improved overall survival in patients who received NACT. However, limited data is available on the South Indian population regarding the same. We aimed to study the short-term outcomes of NACT in locally advanced carcinoma stomach in our institute.

## Materials and methods

This was a single-center, prospective study conducted in the Department of Surgery in collaboration with the Departments of Medical Oncology, Radiodiagnosis, and Pathology at the Jawaharlal Institute of Postgraduate Medical Education and Research, Puducherry, which is a tertiary care teaching hospital in South India, between September 2017 and June 2019. Before the study was initiated, approval was obtained from the Institute Ethics Committee of the institute (study reference number-JIP/IEC/2017/0290). This study was also registered with the Clinical Trial Registry of India with the registration number CTRI/2019/01/016901. Written and informed consent was taken from all the participants and patients were given full freedom to withdraw at any point during the study. Our primary objectives were to study the down-staging effect of NACT on the Tumor Node Metastasis (TNM) stage and to study the R0 resection rate with and without NACT. The secondary objective was to study intraoperative and postoperative outcomes with and without NACT.

All patients aged 18 years and above with histologically proven adenocarcinoma of the stomach diagnosed by esophagogastroduodenoscopy and biopsy, with stages of T2, T3, T4, any N determined by contrast-enhanced computed tomography (CECT) were assessed for eligibility. The patients excluded were aged more than 80 years, having other coexisting malignancy, distant metastasis, recurrent tumors, and Siewert Type I, II gastroesophageal junction tumors. The decision on whether the patient received NACT followed by surgery or upfront surgery was decided by a multidisciplinary tumor board and they were divided into two groups. Patients in Group I were started on neoadjuvant chemotherapy with EOX regime (Injection epirubicin 50 mg/m2 on day one, Injection oxaliplatin 130 mg/m2 on day one, oral capecitabine 625 mg/m2 on days one to 21). CECT thorax, abdomen, and pelvis was done following NACT of three to four cycles to assess response and post-chemotherapy stage of the tumor. Patients underwent subtotal, total, or transhiatal esophago-gastrectomy with D1 plus or D2 lymphadenectomy based on the location of the tumor, and the specimen was sent for histopathological examination. Patients in Group II underwent upfront surgery.

Parameters including age, sex, comorbidities, tumor location, tumor size, TNM stage, type of surgery, duration of surgery, calculated blood loss, duration of hospital stay, and complications were compared between the two groups. Resection completeness, number of harvested and positive lymph nodes, and lymph node ratio (LNR) were studied in the histopathological specimen. Pre and post NACT stage and response to NACT were assessed using Response Evaluation Criteria in Solid Tumours 1.1 (RECIST) criteria in Group I.

The independent variables studied were age, sex, comorbidities, tumor location, Lauren subtype, type of gastrectomy, and extent of lymphadenectomy. The outcome variables studied in Group 1 were pre and post NACT TNM stage and response to NACT by RECIST criteria. Between Groups I and II, R0 resection rates, number of lymph nodes dissected and metastatic nodes, lymph node ratio, duration of surgery, intraoperative blood loss, postoperative complications, and duration of postoperative hospital stay were compared and analyzed.

Statistical analysis was done using SPSS 19.0 software version for Windows (IBM Corp., Armonk, NY, USA). The sample size was calculated to be 68 in each group with an estimated alpha error of 5%, power of 80% and a 12% mean difference in R0 resection rates between the two groups [6]. Continuous variables were analyzed with student T-test and Mann Whitney U test as appropriate. Categorical variables were analyzed with the Chi-Square test and Fisher’s exact test as appropriate. Ordinal data such as the TNM stage of the tumor before and after NACT in Group I was compared using the Wilcoxon signed-rank test. P values were derived from 2-sided tests and a value less than 0.05 was considered statistically significant.

## Results

**Figure 1:**
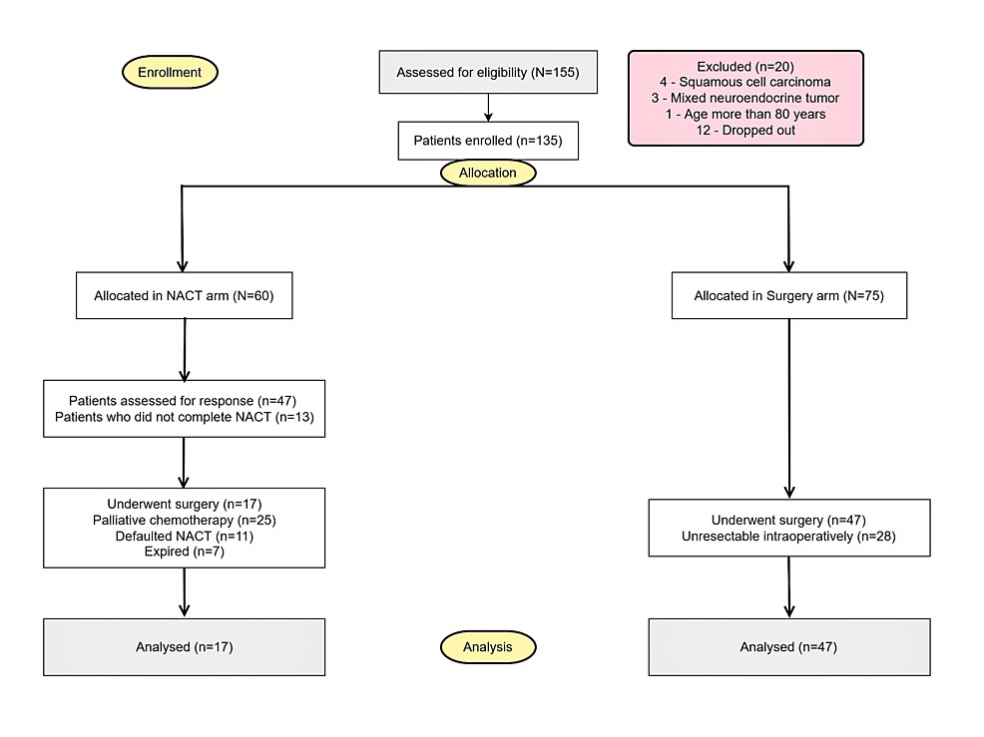
Modified CONSORT flow diagram CONSORT - Consolidated Standards of Reporting Trials NACT - Neoadjuvant Chemotherapy

Between September 2017 and June 2019, 155 patients were assessed for eligibility, out of which 20 patients were excluded from the study (Figure 1).

The remaining 135 patients were allocated into two groups, Group I with 60 patients who received neoadjuvant chemotherapy (NACT arm) and Group II with 75 patients who underwent surgery alone (Surgery arm). Forty-seven patients from the NACT arm were followed up for response assessment CECT. Seventeen patients from the NACT arm and 47 patients in the surgery arm underwent curative surgery. Twenty-eight patients in the surgery arm were deemed unresectable intra-operatively and underwent palliative resection.

Patient and tumor characteristics

Demographic characteristics were comparable between the two groups (Table 1).

**Table 1:** Summary of demographic and patient characteristics between the NACT and Surgery arms. SD - Standard Deviation NACT - Neoadjuvant Chemotherapy * Mann Whitney U test # Chi-Square test ** Fisher Exact test

Demographic Characteristic	NACT and Surgery (n=47)	Surgery alone (n=47)	Total (n=94)	p-value
Age (years)				
Mean ± SD	55 ± 12	55 ± 12	55 ± 12	0.96^*^
Sex				
Male	31 (65.9%)	30 (63.8%)	61 (64.9%)	0.51^#^
Female	16 (34.1%)	17 (36.2%)	33 (35.1%)	
Comorbidities				
Hypertension	2 (4.2%)	8 (17.0%)	10 (10.6%)	0.04^**^
Diabetes Mellitus	1 (2.1%)	5 (10.6%)	6 (6.3%)	
Both Diabetes Mellitus and Hypertension	1 (2.1%)	2 (4.3%)	3 (3.2%)	
Hepatitis B	2 (4.2%)	0	2 (2.1%)	
Addison’s disease	1 (2.1%)	0	1 (1.1%)	
Gastric Outlet Obstruction	8 (17.3%)	27 (57.4%)	35 (37.2%)	<0.001^#^
Site				
Cardia	13 (27.4%)	0	13 (13.8%)	0.002^**^
Body	6 (12.7%)	8 (17%)	14 (14.8%)	
Antropyloric	22 (47.3%)	35 (74.5%)	57 (60.8%)	
Cardia and body	1 (2.1%)	1 (2.1%)	2 (2.1%)	
Body and antrum	2 (4.2%)	3 (6.4%)	5 (5.3%)	
Cardia to antrum	3 (6.3%)	0	3 (3.2%)	
Histological type				
Intestinal	37 (78.7%)	29 (61.7%)	66 (70.2%)	0.06^**^
Diffuse	10 (22.3%)	16 (34%)	26 (27.7%)	
Mixed	0	2 (4.3%)	2 (2.1%)	
T stage				
T2	1 (2.1%)	1 (2.1%)	2 (2.1%)	0.001^**^
T3	13 (27.6%)	25 (53.2%)	38 (40.5%)	
T4a	25 (53.3%)	21 (44.7%)	46 (48.9%)	
T4b	8 (17%)	0	8 (8.5%)	
N stage				
N0	6 (12.7%)	3 (6.4%)	9 (9.5%)	0.39^**^
N1	1 (2.1%)	2 (4.2%)	3 (3.2%)	
N2	28 (59.7%)	21 (44.7%)	49 (52.1%)	
N3	12 (25.5%)	21 (44.7%)	33 (35.2%)	

The anatomical location of the tumor was heterogeneous between the groups. The site of the tumor was represented based on the anatomical location into the cardia, body, antropyloric region. If two or more anatomical locations were involved, they were represented accordingly as mentioned (Table 1). One-fourth of patients who received NACT had the tumor located in the cardia (26.7%). Thirty-five patients (74.5%) in the surgery arm had the tumor located in the antropyloric region compared to 27 patients (45%) in the NACT arm.

During the initial pre-therapeutic evaluation, there was a significant difference in the T stage between the two groups (p = 0.001). T3 tumors were more in the surgery arm, 25 (53.2%) compared to 13 (21.7%) in the NACT arm. All 12 patients (20%) with T4b disease underwent NACT. T2 and T3 tumors combined, which are considered operable, were higher in the surgery arm compared to the NACT arm (53.2% vs 21.7%). T4a and T4b tumors combined were more in the NACT arm compared to the surgery arm (75% vs 44.7%). The pre-therapeutic N stage was comparable between the two groups (p = 0.39). Most patients belonged to N2 and N3 categories in the two groups. N2 included 34 patients (56.7%) in the NACT arm and 21 patients (44.7%) in the surgery arm. N3 included 19 patients (31.7%) in the NACT arm and 21 patients (44.7%) in the surgery arm.

The mean tumor length was 6.8±2.8 cm in the NACT arm and 5.5±2.0 cm in the surgery arm, and a statistically significant difference was noted between the groups (p = 0.01). The mean tumor thickness was 1.9±0.9 cm in the NACT arm and 1.5±0.5cm in the surgery arm and was statistically significant (p = 0.002).

Neoadjuvant chemotherapy arm

Sixty patients received NACT with epirubicin, oxaliplatin, and capecitabine. Eight patients had gastric outlet obstruction and they underwent gastrojejunostomy followed by NACT and were assessed for operability later. The median number of cycles received was six (interquartile range [IQR] 4-7). Tumors that were unresectable based on CECT taken after three cycles were given additional cycles of NACT. Forty-seven patients underwent response assessment CECT after NACT, of which 20 patients were taken up for surgery. Three patients among the 20 were found unresectable intraoperatively and received palliative chemotherapy. The remaining 17 patients underwent transhiatal esophagectomy, total gastrectomy, or distal gastrectomy as appropriate. The NACT completion rate was 78.3%.

Surgical and pathological results

Four patients (23.5%) underwent transhiatal esophago-gastrectomy in the NACT arm. Thirty-nine (83%) patients in the surgery arm underwent distal gastrectomy, compared to six (35.3%) in the NACT arm. Seven patients (41.2%) in the NACT arm underwent total gastrectomy compared to eight patients (17%) in the surgery arm. The proportion of patients who underwent curative resection was 36% in the NACT arm compared to 62.6% in the surgery arm. We found no significant difference in the rate of R0 resection between the groups (p = 0.55). Fifteen patients (88.2%) in the NACT arm underwent R0 resection compared to 40 patients (85.1%) in the surgery arm. One patient (5.9%) underwent R1 resection in the NACT arm compared to seven patients (14.9%) in the surgery arm. The median duration of surgery was seven (IQR 6-13) hours in the NACT group and six (IQR 3-8) hours in the surgery group (p = 0.02). The median blood loss in the NACT arm was 400 ml (IQR 400-550) compared to 360 ml (IQR 350-500) in the surgery arm (p <0.001). Two patients (11.8%) in the NACT arm developed surgical site infection compared to seven (14.9%) in the surgery arm. Two patients (11.8%) developed an anastomotic leak in the NACT arm which was managed conservatively. The complication rate was not statistically significant. We found the NACT arm had significantly fewer lymph nodes harvested (seven in NACT arm vs 19 in surgery arm, p = 0.01) and fewer pathologically positive nodes (one in NACT arm vs three in surgery arm, p = 0.01) compared to the surgery arm, however, the median lymph node ratio was not statistically significant (0.18 in NACT arm vs 0.23 in surgery arm, p = 0.5). We also found that the median days of postoperative hospital stay in the NACT arm was significantly more compared to the surgery arm (10 in NACT vs nine in Surgery, p = 0.01) (Table 2).

**Table 2:** Summary of operative and pathological characteristics between the NACT and the Surgery arms IQR - Interquartile Range NA - Not applicable NACT - Neoadjuvant Chemotherapy SSI - Surgical site infection **Fisher Exact Test *Mann Whitney U test

Parameter	NACT and Surgery (n=17)	Surgery alone (n=47)	p-value	Total
Type of surgery				
Distal Gastrectomy	6 (35.3%)	39 (83%)	<0.001^**^	45 (70.3%)
Total Gastrectomy	7 (41.2%)	8 (17%)		15 (23.4%)
Transhiatal Esophagectomy	4 (23.5 %)	0		4 (6.3%)
Lymphadenectomy				
D1 plus	5 (29.4%)	9 (19.1%)	0.38^**^	14 (21.9%)
D2	12 (70.6%)	38 (80.9%)		50 (78.1%)
Extent of resection				
R0	15 (88.2%)	40 (85.1%)	0.55^**^	55 (85.9%)
R1	1 (5.9%)	7 (14.9%)	0.67^**^	8 (12.5%)
R2	1 (5.9%)	0	0.26^**^	1 (1.6%)
Duration of surgery (days)				
Median	7	6	0.02*	NA
Range	6-13	3-8		
Blood loss (ml)				
Median	400	360	<0.001^*^	NA
IQR	400-550	350-500		
Nodes harvested				
Median	7	19	0.01^*^	NA
IQR	5-17	13-25		
Pathological nodes				
Median	1	3	0.01*	NA
IQR	0-3	0-7		
Lymph node ratio	0.18	0.23	0.5*	NA
Complications				
SSI	2 (11.8%)	7 (14.9%)	0.55^**^	9 (14.1%)
Anastomotic leak	2 (11.8%)	0	0.07^**^	2 (3.1%)
Postoperative hospital stay (days)				
Median	10	9	0.01^*^	NA
IQR	9-15	8-10		

Response to neoadjuvant chemotherapy

**Figure 2:**
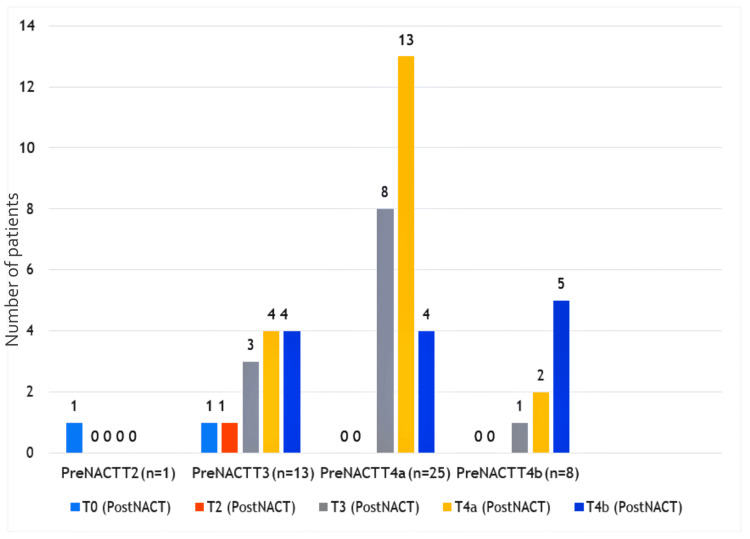
Comparison of T stage before (pre) NACT and the corresponding T stage after (post) NACT on response evaluation. NACT - Neoadjuvant Chemotherapy

We evaluated response to NACT using standard RECIST 1.1 criteria (102). Out of 47 patients, complete response was seen in two patients (4.2%), 13 had a partial response (27.7%), 10 had stable disease (21.3%) and 22 patients had progressive disease (46.8%). There was no significant difference in the down-staging effect of NACT on the T stage (p = 0.88) (Figure 2).

**Figure 3:**
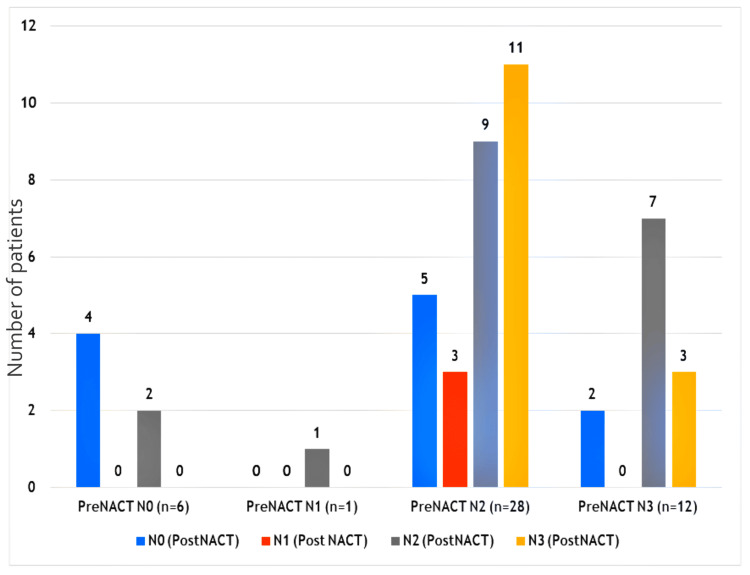
Comparison of N stage before (pre) NACT and the corresponding N stage after (post) NACT on response evaluation. NACT - Neoadjuvant Chemotherapy

Among T2 and T3 tumors in the NACT arm, eight patients out of 14 patients (57%) progressed to T4a and T4b making them inoperable. We did not find a significant difference in the down-staging effect of NACT on the N stage (p = 0.23) (Figure 3).

However, the number of patients who progressed from M0 to M1 during the course of NACT was eight out of 47 patients and was statistically significant (p = 0.01). The demographic and tumor characteristics among patients who were assessed for response to chemotherapy were studied as shown in Table 3.

**Table 3:** Summary of the demographic and tumor characteristics at diagnosis among patients who received neoadjuvant chemotherapy (n=47) SD - Standard deviation

Characteristics	Complete Response (n=2)	Partial Response (n=13)	Stable Disease (n=10)	Progressive Disease (n=22)
Age (years)				
Mean ± SD	47 ± 19	46 ± 9	53 ± 7	53 ± 11
Sex				
Male	1 (50%)	8 (61.5%)	6 (60%)	16 (72.7%)
Female	1 (50%)	5 (38.5%)	4 (40%)	6 (27.3%)
Comorbidities				
Diabetes Mellitus	0	1 (7.7%)	0	0
Hypertension	0	2 (15.4%)	0	0
Both Diabetes Mellitus and Hypertension	0	1 (7.7%)	0	0
Hepatitis B	0	1 (7.7)	0	1 (4.5%)
Site of tumor				
Cardia	1 (50%)	6 (46.2%)	1 (10%)	5 (22.8%)
Body	1 (50%)	2 (15.4%)	1 (10%)	2 (9.1%)
Antropyloric	0	5 (38.4%)	6 (60%)	11 (50%)
Cardia and Body	0	0	0 (10%)	1 (4.5%)
Body and Antrum	0	0	1 (10%)	1 (4.5%)
Cardia to Antrum	0	0	1 (10%)	2 (9.1%)
Histological type				
Intestinal	2 (100%)	11 (84.6%)	9 (90%)	15 (68.2%)
Diffuse	0	2 (15.4%)	1 (10%)	7 (31.8%)
T Stage				
T2	1 (50%)	0	0	0
T3	1 (50%)	4 (30.8%)	1 (10%)	7 (31.8%)
T4a	0	7 (53.8%)	7 (70%)	11 (50%)
T4b	0	2 (15.4%)	2 (20%)	4 (18.2%)
N Stage				
N0	0	1 (7.6%)	3 (30%)	2 (9.1%)
N1	0	0	1 (10%)	0
N2	2(100%)	6 (46.2%)	5 (50%)	15 (68.2%)
N3	0	6 (46.2%)	1 (10%)	5 (22.7%)

Proximally located tumors had a better response to chemotherapy compared to distal. A higher proportion of tumors in patients who had a progressive disease (PD) were of the diffuse type (31.8%).

Follow up of patients

Patients were followed up three months after surgery. In the NACT arm, 17 patients underwent surgery and received adjuvant chemotherapy, 25 received palliative chemotherapy, 11 patients defaulted NACT and were lost to follow up and seven patients expired due to the disease. In the surgery arm, two completed treatment, 31 received adjuvant chemotherapy, and 14 patients received adjuvant chemotherapy elsewhere.

## Discussion

In our study, complete response (CR) to NACT was seen in two patients (4.2%), 13 patients (27.7%) had partial response (PR), 10 patients (21.3%) had stable disease (SD) and 22 patients (46.8%) had PD following NACT as per RECIST 1.1. In a study conducted by Achilli et al., 3% had CR, 34% had PR, 58% had SD and 5% of patients had PD. The number of PD was more in our study while patients with CR and PR were similar [12]. In our study, contrast-enhanced computed tomography (CECT) alone is limited by its ability to accurately differentiate between fibrosis secondary to NACT and residual malignancy [13]. Endoscopic ultrasound and diagnostic laparoscopy were not used for staging in our study, due to resource limitations. Tumor biology, a higher proportion of T4a and T4b disease in the NACT group, poor compliance to chemotherapy due to logistic reasons in the patient population may be responsible for disease progression. The MAGIC trial in the United Kindom and the trial conducted by the Fédération Nationale des Centres de Lutte contre le Cancer (FNLCC) and Fédération Francophone de Cancérologie Digestive (FFCD) in France, have shown better outcomes of NACT with GE junction tumors in concordance with our study [11,14].

The rates of D2 lymphadenectomy performed were comparable in two groups (70.6% in the NACT arm and 80.9% in the surgery arm, p = 0.38). This was better compared to the MAGIC trial where the rates of D2 lymphadenectomy done were 42.5% in the perioperative chemotherapy arm and 40.4% in the surgery arm. Schuhmacher et al. had improved D2 lymphadenectomies done in their study with 95.7% in the chemotherapy group and 92.6% in the surgery group [6]. 

The R0 resection rate was higher in the NACT arm compared to the surgery arm (88.2% vs 85.1%) but was not statistically significant (p = 0.55). Ramachandra et al. reported 87% in the surgery arm and 96% in the chemotherapy arm with no statistical significance (p = 0.33) [15]. Schuhmacher et al. reported a statistically significant R0 resection rate (Z test, p = 0.036) of 81.9% in the chemotherapy arm compared to 66.7% in the surgery arm [6]. Cunningham et al. in their MAGIC trial reported more R0 resection rate in the perioperative chemotherapy group 79.3% compared to 70.3% in the surgery group [11]. A significant R0 resection rate was also reported in the FNCLCC and FFCD Multicenter Phase III trial by Ychou et al. [8]. In a meta-analysis by Xu et al., they reported no significant improvement in the R0 resection rate following NACT (62.86% vs 62.99%, p = 0.81) [7].

The duration of surgery, blood loss, and duration of postoperative hospital stay were significantly higher in the NACT arm. This was probably due to the significant amount of tissue fibrosis and desmoplastic reaction triggered by chemotherapy resulting in a longer duration of surgery and blood loss. It may also be attributed to the number of transhiatal esophago-gastrectomies and total gastrectomies performed in the NACT arm.

The median number of nodes harvested and pathologically positive nodes were significantly lower in the NACT arm compared to the surgery arm (p = 0.01). This may be due to the elimination of micrometastasis and the disappearance of tumor-laden nodes during the course of NACT. Improved survival and prognosis are seen in patients with pathological N0 disease [16-18]. In our study, surgery was done by eight different surgeons with varying levels of skill and expertise. Both the 8th edition of the American Joint Committee on Cancer and the NCCN, recommend a minimum of 16 nodes to be examined for adequate dissection. However, multiple factors including surgeon expertise, small nodes on the specimen which are missed by the pathologist, and fibrosis due to neoadjuvant chemotherapy are responsible for inadequate nodes dissected.

The debate on neoadjuvant chemotherapy continues to exist till date, with significant regional differences in the practices worldwide. The Indian Council of Medical Research (ICMR), in their consensus document for gastric cancer, stated the lack of quality evidence on neoadjuvant regimens to guide the standard of care [19]. In the past, the MAGIC and FNCLCC trials have shown survival advantage when neoadjuvant chemotherapy was given, but the quality of surgical resection in these trials remained doubtful. Most patients did not receive a proper D2 lymphadenectomy. But with the positive results from the recently published fluorouracil, leucovorin, oxaliplatin and docetaxel-4 (FLOT4) study from Germany, the neoadjuvant protocol has started to become the standard of care in most Western centers [20]. It showed a median survival of 50 months with the FLOT regimen versus 35 months with the ECF regimen. Though the neoadjuvant treatment has been accepted as the standard in the West, the far-eastern countries, who have the highest incidence of gastric cancers, still advocate that the upfront D2 gastrectomy is their standard of care. The recently published Japanese Gastric Cancer Treatment guidelines have stated that neoadjuvant chemotherapy can be “conditionally recommended”, only in patients having extensive lymph nodal metastasis [21]. A meta-analysis conducted by Reddavid et al. questioned the role of neoadjuvant chemotherapy in carcinoma stomach. They concluded that a thorough nodal clearance in patients with carcinoma stomach had better overall survival compared to those patients with incomplete resections following NACT [22]. Our study had its limitations, being nonrandomized and the non-availability of diagnostic laparoscopy or endoscopic ultrasound for staging. Our population subset included most patients from a low socio-economic background who require immense motivation for chemotherapy and subsequent follow-up visits, which may have led to disease progression and treatment defaults. Although neoadjuvant chemotherapy is the current standard of care in locally advanced carcinoma stomach, our results show its role may be questionable in such a population where definitive surgery gives equivalent results.

## Conclusions

The rate of R0 resection did not significantly improve with NACT. A good response to NACT was seen only in one-third of patients. In view of high progression rates, appropriate selection criteria would be required when NACT is planned for patients with gastric cancer who have a surgically resectable tumor at presentation. We await survival analysis to further validate the role of NACT in patients with locally advanced gastric cancers.
